# Efficacy of ETB-F01, Heat-Killed *Akkermansia muciniphila* Strain EB-AMDK19, in Patients with Respiratory Symptoms: A Multicenter Clinical Trial

**DOI:** 10.3390/nu16234113

**Published:** 2024-11-28

**Authors:** Hyun Woo Lee, Sang-Nam Lee, Jae-Gu Seo, Yemo Koo, Sung-Yoon Kang, Cheon Woong Choi, So-Young Park, Suh-Young Lee, Sung-Ryeol Kim, Joo-Hee Kim, Hye Sook Choi

**Affiliations:** 1 Division of Respiratory and Critical Care, Department of Internal Medicine, Seoul Metropolitan Government-Seoul National University Boramae Medical Center, College of Medicine, Seoul National University, Seoul 07061, Republic of Korea; athrunzara86@snu.ac.kr; 2 Enterobiome Inc., Goyang-si 10326, Republic of Korea; snlee@enterobiome.com (S.-N.L.); jgseo@enterobiome.com (J.-G.S.); ymkoo@enterobiome.com (Y.K.); 3 Division of Pulmonology and Allergy, Department of Internal Medicine, Gachon University Gil Medical Center, Incheon 21565, Republic of Korea; biomedi@hanmail.net; 4 Department of Respiratory, Allergy and Critical Care Medicine, Kyung Hee University Hospital at Gangdong, College of Medicine, Kyung Hee University, Seoul 02447, Republic of Korea; ccwmd@hanmail.net; 5 Department of Internal Medicine, Chung-Ang University Gwangmyeong Hospital, Gwangmyeong 14353, Republic of Korea; raina821563@gmail.com; 6 Division of Allergy and Clinical Immunology, Department of Internal Medicine, Seoul National University Hospital, Seoul 07061, Republic of Korea; myyoung818@gmail.com; 7 Institute of Allergy and Clinical Immunology, Seoul National University Medical Research Center, Seoul 07061, Republic of Korea; 8 Division of Pulmonology, Allergy and Critical Care Medicine, Department of Internal Medicine, Yongin Severance Hospital, College of Medicine, Yonsei University, Seoul 03722, Republic of Korea; sungryeol@yuhs.ac; 9 Division of Pulmonary, Allergy, and Critical Care Medicine, Department of Medicine, Hallym University Sacred Heart Hospital, College of Medicine, Hallym University, Chuncheon 24252, Republic of Korea; luxjhee@gmail.com; 10 Division of Pulmonary, Allergy and Critical Care Medicine, Department of Internal Medicine, Kyung Hee University Hospital, Seoul 03722, Republic of Korea

**Keywords:** microbiota, signs and symptoms, respiratory, symptom assessment, cough, sputum, dyspnea, randomized controlled trial

## Abstract

Respiratory symptoms are prevalent in the general population, and they are associated with a decline in lung function and increased mortality. The gut–lung connection suggests intestinal dysbiosis may impact lung diseases, with *Akkermansia muciniphila* showing promise in regulating extraintestinal diseases. However, its application in patients with respiratory symptoms lacks clinical trial evidence. In this randomized, double-blind trial, ETB-F01, containing heat-killed *A. muciniphila* strain EB-AMDK19, was compared with a placebo in patients experiencing respiratory symptoms for 4 to 12 weeks. The primary outcome was improvement in Breathlessness, Cough, and Sputum Scale (BCSS) score over 12 weeks. Secondary outcomes included lung function, fractional exhaled nitric oxide (FeNO), modified Medical Research Council (mMRC) dyspnea scale, St. George’s Respiratory Questionnaire (SGRQ), and Visual Analog Scale (VAS) score. The primary analysis was performed in the per-protocol set, with a sensitivity analysis in the full analysis set. In the per-protocol population, 68 participants were randomly assigned to the ETB-F01 group and 65 to the placebo group. ETB-F01 had a superior efficacy over placebo in improving BCSS total scores (between-group difference = −0.8 (95% confidence interval, −1.4–−0.3), *p*-value = 0.004). Specifically, there was a significant reduction in BCSS breathlessness and cough domain scores with ETB-F01. While trends toward improvement in lung function were noted, statistical significance was not achieved. No significant differences were observed in FeNO and other symptom scores (mMRC, SGRQ, and VAS). In safety profile, ETB-F01 did not cause any serious adverse events. These results suggest that ETB-F01 is safe and effective for alleviating respiratory symptoms.

## 1. Introduction

Respiratory symptoms, including breathlessness, cough, and sputum, are predominant reasons for hospital visits in the general population. In individuals aged 40 and older, up to 47.5% cross-sectionally reported experiencing at least one respiratory symptom [1]. This prevalence was higher in females, smokers, and those with comorbidities, elevated body mass index, reduced forced expiratory volume in 1 s (FEV_1_), and lower physical activity levels [1]. In terms of acute respiratory symptoms, a quarter of males and a third of females reported at least one cold-related respiratory symptom, most commonly breathlessness, followed by sputum, in the general population [2]. Regarding chronic respiratory symptoms, even among individuals with normal spirometry without previously diagnosed airway diseases, 32% reported experiencing respiratory symptoms [3].

The clinical significance of respiratory symptoms lies in their consistent correlation with patient prognosis. In the general population, respiratory symptoms are linked to reduced FEV_1_ or FEV_1_ decline [4,5]. Moreover, healthy individuals who experienced respiratory symptoms faced an increased risk of hospitalizations and mortality [3]. Each of the symptoms, namely cough, phlegm, and breathlessness, independently contributed to a higher risk of mortality associated with respiratory causes [6]. Furthermore, there is a significant association between an increasing number of respiratory symptoms and a higher risk of mortality [7].

The gut microbiota has become a critical regulator of host health and disease in terms of metabolism and immunity through producing active metabolites such as short-chain fatty acids (SCFAs), vitamins, bile acid derivatives, antimicrobial peptides [8,9]. Among them, *A. muciniphila*, a representative genus in the *Verrucomicrobiota* phylum, has been identified as a Gram-negative, strictly anaerobic, mucin-degrading and short-chain fatty acid (SCFA)-producing commensal bacterium that colonizes in the mucus layer of the human intestine [10]. This bacterium interacts with human intestinal cells, contributing to the regulation of intestinal barrier function, the modulation of intestinal immunity, and the modification of intestinal inflammation [11]. The clinical attention paid to *A. muciniphila* stems from evidence suggesting a correlation between its abundance in the gut and several human diseases. A notable decrease in its abundance is linked to cardiometabolic diseases, including obesity, diabetes mellitus, cardiovascular diseases, and non-alcoholic fatty liver disease [12,13,14]. Moreover, abundance of *A. muciniphila* exhibits a negative correlation with the occurrence and severity of disorders involving chronic airway inflammation, such as asthma [15,16]. Thus, *A. muciniphila* has attracted considerable attention in recent years as a candidate for next-generation probiotics (NGPs) that are considered promising for the prevention and treatment of dysbiosis-associated diseases [11]. Indeed, preclinical and clinical studies showed that *A. muciniphila* administration could modulate inflammation, mitochondrial activity, thermogenesis, and the metabolism of lipids and glucose, thereby exerting an impact on metabolic health [11,17]. Although *A. muciniphila* has beneficial effects on the host metabolic and immune responses, maintaining its stability and viability is a technological challenge due to its high sensitivity to oxygen [18,19]. Remarkably, the first clinical study revealed that daily oral administration of pasteurized *A. muciniphila* resulted in enhancements in insulin sensitivity, blood lipid profiles, body weight, and fat mass in the individuals who were overweight or obese [17]. Moreover, Amuc_1100, a heat-stable outer membrane protein of *A. muciniphila*, improves host immune responses and intestinal epithelial barrier function by activating Toll-like receptor 2 (TLR2) and replicates almost all of the beneficial effects of live bacterium [19,20]. Several studies have reported the potential clinical benefits of pasteurized *A. muciniphila* or Amuc_1100, with no issues regarding safety or tolerability [18,19]. Furthermore, we recently demonstrated that the administration of heat-killed (pasteurized) *A. muciniphila* EB-AMDK19 isolated from the feces of healthy Koreans alleviates house dust mite (HDM)-induced allergic airway inflammation and airway hyperresponsiveness (AHR) in mice by suppressing Th2-mediated immune responses [21]. However, there have been no applications in clinical trials of heat-killed *A. muciniphila* EB-AMDK19 for patients with respiratory symptoms.

In this study, we aimed to compare ETB-F01 with placebo in terms of improvement in BCSS score over 12 weeks of treatment.

## 2. Materials and Methods

### 2.1. Study Design

**Figure 1:**
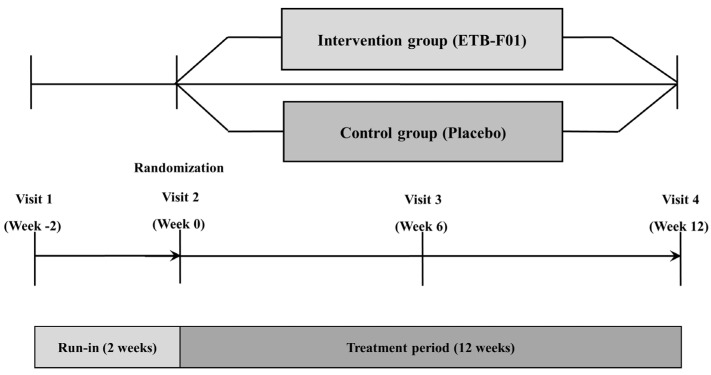
Study design.

This study was a randomized, double-blind, parallel-group, multicenter trial conducted at 9 medical centers in South Korea (Figure 1). Patients meeting the inclusion and exclusion criteria were prohibited from using systemic corticosteroids, immunosuppressants, mucolytics or muco-active agents, any medications for asthma or chronic obstructive pulmonary disease (COPD), gastric acid inhibitors, antibiotics, antidiarrheals, probiotics, prebiotics, and probiotic products starting from the screening (visit 1). A random sequence was generated through block randomization allocation codes using the SAS^®^ program. Upon visit 2, randomization was performed in a 1:1 ratio using central allocation, and neither research staff nor patients were aware of the treatment assignment before or after randomization. Randomization data were strictly confidential until unblinding, accessible only in unavoidable circumstances such as a serious adverse drug reaction or other significant clinical situations.

### 2.2. Patients

Eligible patients were adults aged 19 to 70 years, experiencing two or more of the following symptoms (cough, sputum, or shortness of breath/chest tightness) for a duration of 4 to 12 weeks. Inclusion criteria were (1) a Breathlessness, Cough, and Sputum Scale (BCSS) score of 3 or above but less than 9 and (2) an FEV_1_/FVC ratio of 70% or higher. All patients provided written informed consent for participation before undergoing any study-related procedure. Key exclusion criteria included patients with clinically significant findings in chest X-ray, those diagnosed with or currently under medication for asthma or COPD, patients with chronic bronchitis (i.e., a consistent BCSS score of 9 or above for at least 2 years, with persistent cough and sputum symptoms lasting at least 3 months annually), those diagnosed with respiratory infections caused by viruses or bacteria within the last four weeks, and current smokers or individuals who quit smoking or vaping less than six months ago. Of note, shortness of breath and/or chest tightness were not attributable to cardiovascular diseases in our study population. A complete list of inclusion and exclusion criteria is presented in Table S1.

### 2.3. Procedures

After randomization, patients were administered either ETB-F01 or a placebo for a duration of 12 weeks. The treatment involved daily intake with water of one 500 mg capsule per dose. ETB-F01 is composed of heat-killed *A*. *muciniphila* EB-AMDK19, encapsulated at a concentration of 5.0 × 10^10^ cells. The placebo included corn starch and maltodextrin in the exact shape of the ETB-01 capsule. On the morning of the randomization visit (visit 2), baseline data were collected for BCSS, pre-bronchodilator spirometry FEV_1_, forced vital capacity (FVC), FEV_1_/FVC ratio, fractional exhaled nitric oxide (FeNO), modified Medical Research Council (mMRC) dyspnea scale score, St. George’s Respiratory Questionnaire (SGRQ) score, and Visual Analog Scale (VAS) score. Upon each subsequent visit (visit 3 and 4), BCSS, mMRC, SGRQ, and VAS score data were collected. Data from spirometry and FeNO were collected at the end of the treatment period.

### 2.4. Ethics

This study adhered to the principles outlined in the Declaration of Helsinki, and all enrolled patients granted written informed consent upon study commencement. The study received approval from the institutional review board at Gachon University Gil Hospital (protocol code GCIRB2022-201 and date of approval 21 July 2022), Kyung Hee University Hospital at Gangdong (protocol code KHNMC 2022-07-013 and date of approval 29 August 2022), Kyung Hee University Medical Center (protocol code KHUH 2022-07-056 and date of approval 13 September 2022), Korea University Anam Hospital (protocol code 2022AN0377 and date of approval 8 August 2022), Seoul National University Hospital (protocol code J-2210-002-1364 and date of approval 5 October 2022), SNUH SMG-SNU Boramae Medical Center (protocol code 30-2022-69 and date of approval 25 August 2022), Yonsei University Yongin Severance Hospital (protocol code 9-2022-0104 and date of approval 26 September 2022), Chung-Ang University Gwangmyeong Hospital (protocol code 2207-015-015 and date of approval 17 October 2022), and Hallym University Sacred Heart Hospital (protocol code HALLYM 2022-07-012 and date of approval 25 July 2022), respectively. This study was conducted in compliance with the Declaration of Helsinki and the International Conference on Harmonization Good Clinical Practice. No significant protocol amendments affecting any randomized patients were made. The study protocol was registered with the Clinical Research Information Service of the Republic of Korea (KCT0008680).

### 2.5. Outcomes

The primary efficacy outcome was improvement in BCSS score from baseline to week 12. The BCSS score includes total scores within three domains: breathlessness, cough score, and sputum. The secondary efficacy outcomes were changes from baseline in pre-bronchodilator FEV_1_, FVC, and FEV_1_/FVC ratio; FeNO; mMRC dyspnea scale; SGRQ; and VAS scores for breathlessness, cough, and sputum.

Safety outcomes involved the recording of all adverse events including general disorders, specific disorders, and serious adverse events including cardiac, respiratory, hematologic, and hepatologic abnormality. Blood laboratory tests—including white blood cell (WBC) count, red blood cell (RBC) count, hemoglobin (HB), hematocrit (Hct), platelets, neutrophils, lymphocytes, monocytes, eosinophil, basophil, alanine aminotransferase (ALT), aspartate aminotransferase (AST), total cholesterol, glucose, total protein, blood urea nitrogen (BUN), creatinine, uric acid, and calcium (Ca)—were performed at baseline and 12 weeks. A chest X-ray was allowed at weeks 6 and 12 if pneumonia was suspected. Blood pressure measurements, 12-lead ECGs, and vital signs were assessed at screening and after 6 and 12 weeks of treatment.

### 2.6. Statistical Analysis

Our study employed a superiority test with a significance level of 5% and a two-tailed test, maintaining a power of 80% with a Type II error (β) of 0.2. We aimed for an equal ratio of trial sample sizes between the treatment and placebo groups (1:1). In a previous study using BCSS as primary assessment variable, the treatment group showed a decrease of 1.1 points, compared to 0.1 in the placebo group [22]. The standard deviation was estimated to be 1.0, higher for sample size calculation. Additionally, a conservative approach was taken, applying a mean difference of 0.53 between the two groups. A minimum of 56 participants per group was determined necessary to detect significant differences between the treatment and placebo groups, considering a 25% dropout rate, resulting in a total enrollment of 150 participants to ensure adequate statistical power.

The data were analyzed in three main forms: the safety set, full analysis (FA) set, and per-protocol (PP) set. Efficacy outcomes were primarily analyzed using the PP set, with sensitivity analyses conducted using the FA set. Adverse events were analyzed using the safety set. Between-group comparisons were performed based on the results of the normality test (Shapiro–Wilk). If both treatment and control groups satisfied the assumption of normality (*p*-value > 0.05), a two-sample *t*-test was employed for comparative analysis. In cases in which at least one group did not meet the assumption of normality, the Wilcoxon rank sum test was utilized for comparative analysis. If there was a statistically and clinically significant difference in demographic information between groups, an ANCOVA could be performed using the relevant characteristics as covariables. Significance testing for differences was conducted using two-tailed tests with a significance level of *p*-value < 0.05. Statistical analyses were performed using SAS^®^ (Version 9.4, SAS Institute, Cary, NC, USA).

## 3. Results

**Figure 2:**
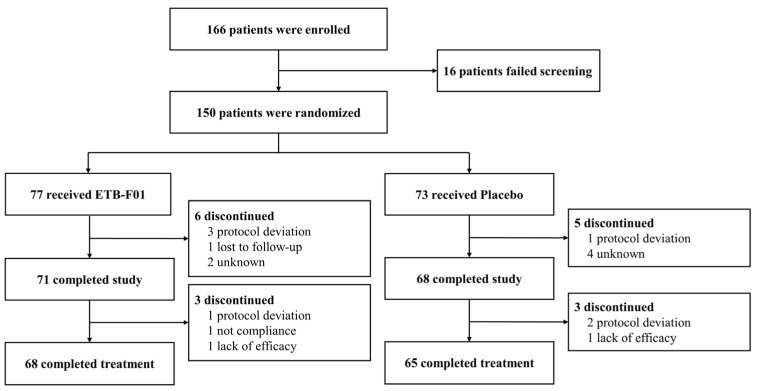
Flow diagram for inclusion and exclusion.

We recruited 166 patients between 1 December 2022 and 18 August 2023, of whom 150 were randomly assigned, with 68 (88%) of 77 completing treatment in the ETB-F01 group and 65 (89%) of 73 in the placebo group (Figure 2). Of note, none of the included patients suffered an ongoing COVID-19 infection or presented with post-COVID symptoms. Compliance with treatment was high, with a mean of 96.0 (±9.0) % in the ETB-F01 group and 95.2 (±9.2) % in the placebo group, without statistical significance.

### 3.1. Baseline Characteristics

In the per-protocol population, there were no clinically significant differences in demographic characteristics, symptoms, lung function, FeNO, and comorbidities between the two groups, except previous smoking history (*p*-value = 0.026, Table 1). The majority of patients (76%) were female, with a mean age of 41.2 years. On trial entry, the baseline BCSS score was 4.4, comprising a breathlessness score of 1.1, a cough score of 1.9, and a sputum score of 1.5. The SGRQ score stood at 30.0, and mMRC was 1.1. Upon VAS assessment, breathlessness was 24.7 mm, cough was 32.7 mm, and sputum was 36.4 mm. Mean values for FEV_1_, FVC, and FEV_1_/FVC were 3.0 L, 3.7 L, and 82.0%, respectively.

### 3.2. BCSS

ETB-F01 demonstrated superior efficacy over the placebo in improving the BCSS total score from baseline to week 12 (−2.9 (±1.6) vs. −2.0 (±1.6), *p*-value = 0.004, as shown in Table 2 and Figure S1). Consistent results were found in the FA set (−2.7 (±1.8) vs. −2.1 (±1.8), *p*-value = 0.023, Figure S2). The LS mean change from baseline to week 12 in the BCSS total score was −2.4 (±0.4) for ETB-F01 and −1.5 (±0.4) for the placebo, with an adjusted *p*-value of 0.005 (Table 2).

In terms of the BCSS breathlessness domain, ETB-F01 was superior to the placebo in mean changes from baseline to week 12 (−0.7 (±0.8) vs. −0.4 (±0.8), *p*-value = 0.009, as shown in Table 2 and Figure S3). Similar results were found in the FA set (−0.7 (±0.8) vs. −0.4 (±0.8), *p*-value = 0.018, Figure S4). The LS mean change values from baseline to week 12 were −0.5 (±0.2) for ETB-F01 and −0.2 (±0.2) for the placebo, with an adjusted *p*-value of 0.011.

Regarding BCSS cough scores, ETB-F01 showed a significant improvement from baseline to week 12 (−1.3 (±0.8) vs. −0.9 (±0.9), *p*-value = 0.011, Table 2 and Figure S5). However, no significant differences were observed in the FA set (Figure S6). The LS mean change values were −0.8 (±0.2) for ETB-F01 and −0.5 (±0.2) for the placebo, with an adjusted *p*-value of 0.020.

No significant difference was observed in the changes of BCSS sputum scores from baseline to week 12 between the two groups (Figures S7 and S8).

### 3.3. Lung Function and FeNO

**Figure 3:**
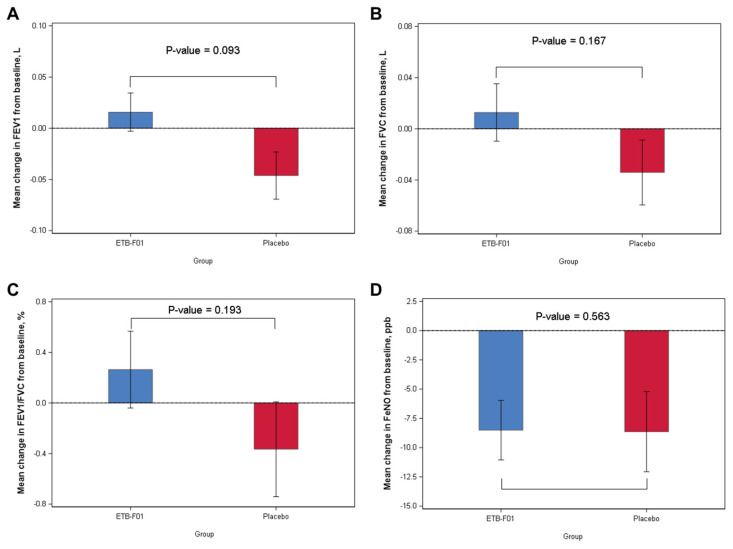
Comparison of the mean changes from baseline to 12 weeks in FEV_1_ (**A**), FVC (**B**), FEV_1_/FVC (**C**), and FeNO (**D**) between the ETB-F01 and the placebo groups. The differences between the two groups were analyzed using two sample *t*-tests. The error bars represent mean  ±  standard deviation (SD). FEV_1_: forced expiratory volume in the first second; FVC: forced vital capacity; FeNO: fractional exhaled nitric oxide.

For the spirometric outcomes of the mean change from baseline to week 12 in FEV_1_, FVC, and FEV_1_/FVC, the differences between the ETB-F01 and the placebo groups were not statistically significant (Figure 3). Additionally, the mean change in FeNO from baseline to week 12 was not statistically significant between the two groups.

### 3.4. Analyses of Other Outcomes

ETB-F01 did not show superior efficacy over the placebo in improving the mMRC and SGRQ from baseline to week 12 (Table S2). In the analyses of VAS with the three domains of breathlessness, cough, and sputum, ETB-F01 did not show statistically significant improvements from baseline to week 12 compared to the placebo (Table S3).

### 3.5. Safety Profiles

In the ETB-F01 group, a total of 12 (15.58%) patients experienced 13 adverse events, while in the control group, 14 patients experienced 22 adverse events. No serious adverse events occurred in either group. There were no statistically significant differences in adverse event profiles between the two groups (Table 3). Additionally, the values for hematologic and blood biochemical parameters were within the normal range both before and after the intervention, and all parameters showed no statistically significant differences within and between the ETB-F01 and the placebo groups (Table S4).

## 4. Discussion

The concept of the gut–lung connection stems from growing evidence suggesting that intestinal dysbiosis can impact the onset or severity of lung diseases [23]. Remarkably, children with diagnosed allergic asthma showed lower levels of *A*. *muciniphila* compared to their healthy controls [15]. Infants with a reduced abundance of *A*. *muciniphila* faced an elevated risk of developing asthma symptoms in later stages of life [24]. Moreover, a lower abundance of *A*. *muciniphila* was associated with a higher severity of asthma in adults [16]. *Michalovich* et al. demonstrated that the oral administration of live *A. muciniphila* significantly reduces airway inflammation in an allergen-induced allergic asthma mouse model [16], suggesting its potential utility in asthma. Importantly, our latest study revealed that heat-killed *A*. *muciniphila* EB-AMDK19 administration mitigated HDM-induced allergic airway inflammation and AHR in mice by suppressing Th2-mediated immune responses [21]. However, evaluations of the efficacy and safety of *A. muciniphila* have been based mainly on mice, with those involving humans being rare. Our study is an inaugural clinical trial of *A. muciniphila* supplementation targeting respiratory conditions. Herein, we provide novel evidence on the potential effects of ETB-F01, a formulation integrating heat-killed *A*. *muciniphila* EB-AMDK19, on ameliorating symptoms of patients experiencing respiratory symptoms for 4 to 12 weeks without significant adverse events.

We employed a PP analysis to evaluate the potential maximum benefit of ETB-F01, complemented by a robustness-confirming sensitivity analysis using the FA set, supported by comparable dropout rates and baseline characteristics across study groups. Our primary outcome, i.e., improvement in BCSS total scores over the 12-week treatment period, demonstrated the superior efficacy of ETB-F01 over the placebo. ETB-F01 showed a moderately effective reduction of 0.8 points in total BCSS score, surpassing the minimum clinically important difference threshold when compared to the placebo [25]. These findings are consistent in both the PP and FA set, underscoring the robustness of our results. Among the three domains of BCSS, breathlessness and cough were significantly improved with ETB-F01, but not sputum. Based on these findings, it is speculated that ETB-F01, rather than directly influencing excessive mucus production or impaired clearance, may mitigate AHR and hypersensitivity of the cough reflex associated with bronchial inflammation. Indeed, there was a trend toward FEV_1_ improvement in the ETB-F01 group, but statistical significance was not reached. In support of this, our previous study showed that heat-killed *A. muciniphila* EB-AMDK19 administration alleviates HDM-induced AHR and airway inflammation in mice [21]. Moreover, although secondary outcomes such as mMRC and SGRQ did not exhibit significant differences between the two groups, the improvement in VAS scores from baseline to week 12 appeared more pronounced in the ETB-F01 group, albeit without statistical significance. It is noteworthy that responses to VAS showed wider variability in both groups, indicating the more subjective nature of responses compared to BCSS.

Our study focused on individuals presenting respiratory symptoms such as breathlessness, cough, or sputum persisting for a duration of 4 to 12 weeks. Currently, a standardized definition for this specific patient subgroup is lacking, and epidemiological investigations dedicated to this subgroup are also scarce. Given that cough was a symptom in over 99% of our patients, the etiology and pathogenesis of the respiratory symptoms with a subacute course can be inferred through previous studies on subacute cough. Typically, patients with subacute cough undergo spontaneous remission within 8 weeks without treatment, predominantly associated with post-infectious cough [26]. However, compared to the placebo group, a significantly greater improvement in BCSS at 12 weeks was observed in the ETB-F01 group, suggesting the potential clinical benefit of ETB-F01 in reducing the duration of respiratory symptoms in patients. In our study, patients with underlying lung diseases such as asthma and COPD were excluded, and those with chronic rhinitis made up only 4.5%, indicating that post-infectious cough was likely the primary cause. The exact pathogenesis of post-infectious cough remains elusive, with theories suggesting it arises from extensive disruption of integrity in airway epithelium and widespread mucosal inflammation involving the upper or lower airways, occasionally accompanied by transient AHR [27]. Notably, the majority of our patients reported breathlessness, with an average BCSS breathless score of 1 and VAS breathlessness of 25 mm. Therefore, it can be hypothesized that ETB-F01’s beneficial effects on respiratory symptoms may have a role in attenuating airway inflammation in a symptomatic population without previous airway disease.

Despite growing interest in *A. muciniphila* over the past decade as a promising NGP [11], the specific mechanisms by which *A. muciniphila* provides beneficial effects on respiratory symptoms remain poorly understood. Following the gut–lung axis hypothesis, it has been suggested that gut microbiota-derived SCFAs have the potential to enhance lung immunity or modify excessive inflammation [23]. Indeed, many studies underscore the immunomodulatory functions of SCFAs in intestinal epithelial and innate immune cells through inhibition of histone deacetylases (HDACs) and activation of G-protein-coupled receptors (GPRs) like GPR41, GPR43, and GPR109A [28,29]. For instance, SCFAs inhibit the production of proinflammatory mediators including nitic oxide (NO), TNF-α, and IL-6 and enhance the production of a key anti-inflammatory cytokine IL-10 by inhibiting HDAC activity in macrophages [29]. Additionally, the SCFA butyrate suppresses oxidative stress and induces epithelial tight junction expression by activating GPR in the colonic mucosa, thereby improving inflammation and colonic defense barrier [28]. Remarkably, our previous study revealed that oral administration of heat-killed *A. muciniphila* EB-AMDK19 increased the relative abundance of specific SCFA-associated cecal microbiota in HDM-induced allergic asthma mice [21]. Given these previous findings, it is conceivable that heat-killed *A. muciniphila* EB-AMDK19 supplementation may induce SCFAs by modulation of the gut microbiome, thereby mitigating local and systemic inflammation. The other hypothesis on the correlation between *A. muciniphila* and respiratory symptoms posits a potential connection with immune-modulating cytokines or regulatory T cells (Tregs) present in the bloodstream. *Bian* et al. demonstrated that oral *A. muciniphila* administration exerts a protective effect against dextran sulfate sodium-induced ulcerative colitis in mice by reducing proinflammatory cytokines TNF-α, IL-6, IL-1α, and IL-12A and elevating immunosuppressive cytokine IL-10 in the serum [30]. Moreover, oral administration of *A. muciniphila* to high-fat diet (HFD)-fed mice increases the level of Foxp3^+^ Tregs in the adipose tissue, leading to the attenuation of obesity-induced local and systemic inflammation [31]. Furthermore, our previous study showed that HDM-challenged mice that were administered heat-killed *A. muciniphila* EB-AMDK19 exhibited an increase in the generation of Foxp3^+^ Tregs in the spleen [21]. Given that (i) orally administrated *Lactiplantibacillus plantarum* induces a tolerogenic phenotype of dendritic cells (DCs) in the intestinal lamina propria or Peyer’s patches and then these DCs migrate to the spleen and stimulate the generation of Tregs [32], (ii) tolerogenic DCs induce TLR2-mediated immunosuppressive responses [33], and (iii) the TLR2-interacting heat-stable membrane protein Amuc_1100 of *A. muciniphila* [19,20] reduces sepsis-induced lung histopathological damage and serum pro-inflammatory cytokine and chemokine levels in rats [34], it is possible that TLR2 triggering on activated DCs by Amuc_1100 may contribute to the recruitment, expansion, and function of Foxp3^+^ Tregs in the lung.

The present study has several limitations. First, although the sample size was statistically calculated, this study encountered limitations in sample size, as only 150 patients were randomly assigned, and this number further reduced to 133 patients in the per-protocol population. In addition, we analyzed sex differences in the efficacy of ETB-F01 on respiratory symptoms in the per-protocol population, of whom 101 (55 ETB-F01 and 46 placebo) were females and 32 (13 ETB-F01 and 19 placebo) were males. The mean changes in BCSS total score and individual domains (breathlessness and cough) from baseline to week 12 were statistically significant between the two groups in females, but not in males, possibly reflecting the small sample size (Table S5). It is, however, noteworthy that an improving trend in BCSS was observed with ETB-F01 compared with placebo in males. A more extensive and diverse sample could strengthen the generalizability of the findings, offering a more robust foundation for drawing conclusions regarding the efficacy and safety of ETB-F01. Second, the planned comprehensive analysis using blood and urine samples to elucidate the mechanism of ETB-F01’s action could not be performed because only six patients agreed to contribute samples. Furthermore, as we used only heat-killed *A*. *muciniphila*, the effects of live *A*. *muciniphila* on respiratory conditions remain unknown. This limitation restricts the depth of understanding regarding the potential correlation between *A*. *muciniphila* and immune-modulating cytokines, hindering the exploration of additional mechanistic pathways. Third, we could not find significant differences in the diversity and composition of human fecal microbiota between the ETB-F01 and placebo groups, possibly due to the lack of a standardized definition for the specific subgroup in our study population, the inter-individual variability of gut microbial taxa, and individualized microbiota responses to the altered intestinal microenvironment. Finally, the use of BCSS to determine respiratory symptoms for 4 to 12 weeks may face criticism, as it has been primarily validated for assessing chronic respiratory symptoms in COPD patients [35]. Nevertheless, being a questionnaire that employs a 5-point Likert scale, BCSS is not inherently restricted in its application and can assess symptoms across a spectrum from acute to chronic [25,35,36]. Considering its broad applicability, extending the application of BCSS to patients with respiratory symptoms beyond COPD appears feasible.

## 5. Conclusions

This 12-week double-blind, placebo-controlled clinical trial on the effects of ETB-F01 in patients with respiratory symptoms for 4 to 12 weeks indicated a significant reduction in BCSS total scores, particularly in the breathlessness and cough domains. ETB-F01 did not cause any severe adverse events. Our findings highlight the promising health benefits of ETB-F01, a safe and feasible strategy for the amelioration of respiratory symptoms. Further exploration is warranted to understand underlying mechanisms and enhance the generalizability of findings.

## Figures and Tables

**Table 1:** Baseline characteristics.

	ETB-F01 (*n* = 68)	Placebo (*n* = 65)	*p*-Value
Age, y, mean ± SD	41.1 ± 12.6	41.3 ± 10.6	0.864
Female, *n* (%)	55 (80.9)	46 (70.8)	0.173
Body weight, kg	60.4 ± 12.2	64.3 ± 14.1	0.073
Height, cm	162 ± 8	164 ± 9	0.111
Smoking history			0.026
Never smoker, *n* (%)	68 (100)	60 (92.3)	
Ex-smoker, *n* (%)	0	5 (7.7)	
Patient-reported symptoms			
Breathlessness, *n* (%)	50 (73.5)	42 (64.6)	0.266
Cough, *n* (%)	67 (98.5)	65 (100)	1.000
Sputum, *n* (%)	65 (95.6)	63 (96.9)	1.000
BCSS score, total, mean ± SD	4.4 ± 1.5	4.3 ± 1.3	0.711
Breathlessness score, mean ± SD	1.1 ± 0.7	1.0 ± 0.6	0.248
Cough score, mean ± SD	1.9 ± 0.7	1.8 ± 0.7	0.517
Sputum score, mean ± SD	1.5 ± 0.7	1.5 ± 0.8	0.439
SGRQ score, total, mean ± SD	28.3 ± 18.4	31.7 ± 17.1	0.166
mMRC score, mean ± SD	1.1 ± 0.6	1.2 ± 0.7	0.971
VAS, mm			
Breathlessness, mean ± SD	24.7 ± 22.0	24.7 ± 21.1	0.845
Cough, mean ± SD	32.1 ± 20.6	33.3 ± 20.0	0.690
Sputum, mean ± SD	34.6 ± 21.9	38.2 ± 23.9	0.415
Lung function			
Pre-BDR FEV1, L, mean ± SD	2.9 ± 0.7	3.2 ± 0.8	0.066
Pre-BDR FVC, L, mean ± SD	3.6 ± 0.8	3.9 ± 1.0	0.147
Pre-BDR FEV1/FVC, %, mean ± SD	81.4 ± 6.2	82.6 ± 5.1	0.200
FeNO, ppb	32.8 ± 22.9	34.9 ± 26.1	0.753
Comorbidities			
Hypertension, *n* (%)	1 (1.5)	3 (4.6)	0.358
Diabetes mellitus, *n* (%)	1 (1.5)	0 (0)	1.000
Dyslipidemia, *n* (%)	1 (1.5)	2 (3.1)	0.483
Angina pectoris, *n* (%)	0 (0)	1 (1.5)	0.489
Cerebrovascular disease, *n* (%)	1 (1.5)	0 (0)	1.000
Chronic rhinitis, *n* (%)	4 (5.9)	2 (3.1)	0.885
Gastroesophageal reflux, *n* (%)	0 (0)	1 (1.5)	0.489

BCSS, Breathlessness, Cough, and Sputum Scale; FeNO, fractional exhaled nitric oxide; FEV1, forced expiratory volume in one second; FVC, forced vital capacity; mMRC, Modified Medical Research Council Dyspnea Scale; ppb, parts per billion; Pre-BDR, before bronchodilator; SD, standard deviation; SGRQ, St. George’s Respiratory Questionnaire; VAS, Visual Analog Scale.

**Table 2:** BCSS score.

Outcome	ETB-F01 (*n* = 68)	Placebo (*n* = 65)
**BCSS total score**		
At week 12, mean ± SD	1.6 ± 1.4	2.3 ± 1.6
Change from baseline to week 12, mean ± SD	−2.9 ± 1.6	−2.0 ± 1.6
*p*-value within groups	<0.001	<0.001
*p*-value between groups	0.004	
Change from baseline to week 12, LS mean ± SE	−2.4 ± 0.4	−1.5 ± 0.4
Difference in change from baseline to week 12, LS mean, (95% CI)	−0.8 (−1.4, −0.3)	
*p*-value ^a^	0.005	
**BCSS breathlessness score**		
At week 12, mean ± SD	0.4 ± 0.6	0.6 ± 0.6
Change from baseline to week 12, mean	−0.7 ± 0.8	−0.4 ± 0.8
*p*-value within groups	<0.001	<0.001
*p*-value between groups	0.009	
Change from baseline to week 12, LS mean ± SE	−0.5 ± 0.2	−0.2 ± 0.2
Difference in change from baseline to week 12, LS mean, (95% CI)	−0.4 (−0.6, −0.1)	
*p*-value ^a^	0.011	
**BCSS cough score**		
At week 12, mean ± SD	0.6 ± 0.6	0.9 ± 0.7
Change from baseline to week 12, mean ± SD	−1.3 ± 0.8	−0.9 ± 0.9
*p*-value within groups	<0.001	<0.001
*p*-value between groups	0.011	
Change from baseline to week 12, LS mean ± SE	−0.8 ± 0.2	−0.5 ± 0.2
Difference in change from baseline to week 12, LS mean, (95% CI)	−0.3 (−0.6, −0.1)	
*p*-value ^a^	0.020	
**BCSS sputum score**		
At week 12, mean ± SD	0.6 ± 0.6	0.8 ± 0.7
Change from baseline to week 12, mean ± SD	−0.9 ± 0.7	−0.8 ± 0.8
*p*-value within groups	<0.001	<0.001
*p*-value between groups	0.540	
Change from baseline to week 12, LS mean ± SE	−1.0 ± 0.2	−0.9 ± 0.2
Difference in change from baseline to week 12, LS mean, (95% CI)	−0.1 (−0.4, 0.2)	
*p*-value ^a^	0.380	

BCSS, Breathlessness, Cough, and Sputum Scale; CI, confidence interval; LS, least squares; SD, standard deviation; SE, standard error. ^a^ *p*-value for ANCOVA-adjusted smoking history.

**Table 3:** Adverse events.

	ETB-F01 (*n* = 77)	Placebo (*n* = 73)	*p*-Value
No. of patients with adverse events (%)	12 (15.6)	14 (19.2)	0.357
Pharyngitis	4 (5.2)	2 (2.7)	0.883
Musculoskeletal pain	0 (0)	3 (4.1)	0.113
Leukocytosis	0 (0)	2 (2.7)	0.235
Lymphopenia	0 (0)	2 (2.7)	0.235
Dermatitis	0 (0)	2 (2.7)	0.235
Respiratory disorder	1 (1.3)	0 (0.0)	1.000
Hematuria	1 (1.3)	0 (0.0)	1.000
Agitation	1 (1.3)	0 (0.0)	1.000
Amenorrhea	0 (0)	1 (1.4)	0.487
Dysmenorrhea	0 (0)	1 (1.4)	0.487
Otitis media	0 (0)	1 (1.4)	0.487
Dyspepsia	2 (2.6)	2 (2.7)	0.670
Gastritis	1 (1.3)	0 (0.0)	1.000
No. of patients with serious adverse events (%)	0 (0)	0 (0)	-

## Data Availability

The data used in this research cannot be shared publicly to maintain patient confidentiality and comply with ethical guidelines. However, researchers interested in accessing the data for scientific collaboration or verification purposes may contact the corresponding author and submit a formal request. The release of data will be subject to ethical approval, ensuring that privacy and confidentiality standards are upheld in accordance with institutional policies and regulations.

## Supplementary Materials

The following supporting information can be downloaded at: https://www.mdpi.com/article/10.3390/nu16234113/s1; Table S1. The complete list of inclusion and exclusion criteria; Figure S1. Mean change from baseline in BCSS total score in the per-protocol set; Figure S2. Mean change from baseline in BCSS total score in the full analysis set; Figure S3. Mean change from baseline in BCSS breathlessness score in the per-protocol set; Figure S4. Mean change from baseline in BCSS breathlessness score in the full analysis set; Figure S5. Mean change from baseline in BCSS cough score in the per-protocol set; Figure S6. Mean change from baseline in BCSS cough score in the full analysis set; Figure S7. Mean change from baseline in BCSS sputum score in the per-protocol set; Figure S8. Mean change from baseline in BCSS sputum score in the full analysis set; Table S2. mMRC and SGRQ; Table S3. VAS; Table S4. Blood test results. Table S5. BCSS score according to sex groups.

## Abbreviations

BCSS: Breathlessness, Cough, and Sputum Scale; COPD, chronic obstructive pulmonary disease; FeNO, fractional exhaled nitric oxide; VAS, Visual Analog Scale; mMRC, Modified Medical Research Council; SGRQ, St. George’s Respiratory Questionnaire; ETB-F01, heat-killed *Akkermansia muciniphila* strain EB-AMDK19; SCFAs, short-chain fatty Acids; TNF-α, tumor necrosis factor-alpha, IL-6, interleukin-6; IL-1α, interleukin-1 alpha; IL-12A, interleukin-12A; IL-10, interleukin-10; NF-κB, nuclear factor-kappa B.
