# Supplementary material for: Efficacy of ETB-F01, Heat-Killed Akkermansia muciniphila Strain EB-AMDK19, in Patients with Respiratory Symptoms: A Multicenter Clinical Trial
Source: Nutrients. 2024 Nov 28;16(23):4113. doi: 10.3390/nu16234113 (PMC11643724; doi:10.3390/nu16234113)
Supplement: Supplementary file 1 [file nutrients-16-04113-s001.zip › nutrients-3250102-supplementary.pdf]

# Supplementary Materials

## Efficacy of EB-F01, Heat-Killed *Akkermansia muciniphila* Strain EB-AMDK19, in Patients with Respiratory Symptoms: A Multicenter Clinical Trial

Hyun Woo Lee, Sang-Nam Lee , Jae-Gu Seo, Yemo Koo, Sung-Yoon Kang, Cheonwoong Choi, So-Young Park, Suh-Young Lee, Sung-Ryeol Kim, Joo-Hee Kim and Hye Sook Choi

**Table S1. The complete list of inclusion and exclusion criteria.**

| **Inclusion Criteria** |
|---|
| 1) Adults aged 19 to 70 years<br>2) Presence of two or more of the following symptoms (cough, sputum, or shortness of breath/chest tightness) for one month to less than three months, with a BCSS score of 3 or above but less than 9<br>3) FEV1/FVC ratio of 70% or higher<br>4) Written informed consent for participation provided before the commencement of the trial |
| **Exclusion Criteria** |
| 1) Currently undergoing treatment for severe cardiovascular, immune, gastrointestinal/hepatic/biliary, renal/urinary, neurological, musculoskeletal, psychiatric, infectious diseases, or malignant tumors (individuals with cancer in remission for at least 5 years are eligible)<br>2) Clinically significant respiratory findings in chest X-ray results<br>3) Diagnosis of and current medication treatment for chronic obstructive pulmonary disease or asthma<br>4) Chronic bronchitis (consistent BCSS score of 9 or above for at least 2 years with persistent cough and sputum symptoms lasting at least 3 months annually)<br>5) Respiratory infection diagnosed by viruses or bacteria within the last four weeks<br>6) Use of systemic corticosteroids or immunosuppressants within four weeks prior to visit 1<br>7) Medication adjustment for bronchodilators within one week prior to visit 1<br>8) Current smokers or individuals who quit smoking or vaping less than six months ago<br>9) Alcohol consumption exceeding 30 g/day for males or 20 g/day for females within the last four weeks from visit 1<br>10) Creatinine levels exceeding twice the upper limit of normal<br>11) AST(GOT) or ALT(GPT) levels exceeding three times the upper limit of normal<br>12) Uncontrolled hypertension (systolic blood pressure ≥160 mmHg or diastolic blood pressure ≥100 mmHg, measured after 10 minutes of stability for trial participants)<br>13) Uncontrolled diabetes (fasting blood glucose ≥180 mg/dL)<br>14) Use of antibiotics or antidiarrheals within two weeks prior to visit 1<br>15) Use of probiotics, prebiotics, or regular intake (≥4 times per week) of probiotic products within two weeks prior to visit 1<br>16) Pregnancy, lactation, or planning to become pregnant during the trial<br>17) Sensitivity or allergy to ingredients in the trial-specific food<br>18) Participation in another interventional clinical trial (including human trials) within three months before visit 1 or planning to participate in another interventional clinical trial (including human trials) after the commencement of this trial<br>19) Other reasons deemed by the investigator as unsuitable for participation |

Figure S1. Mean change from baseline in BCSS total score in the per-protocol set.

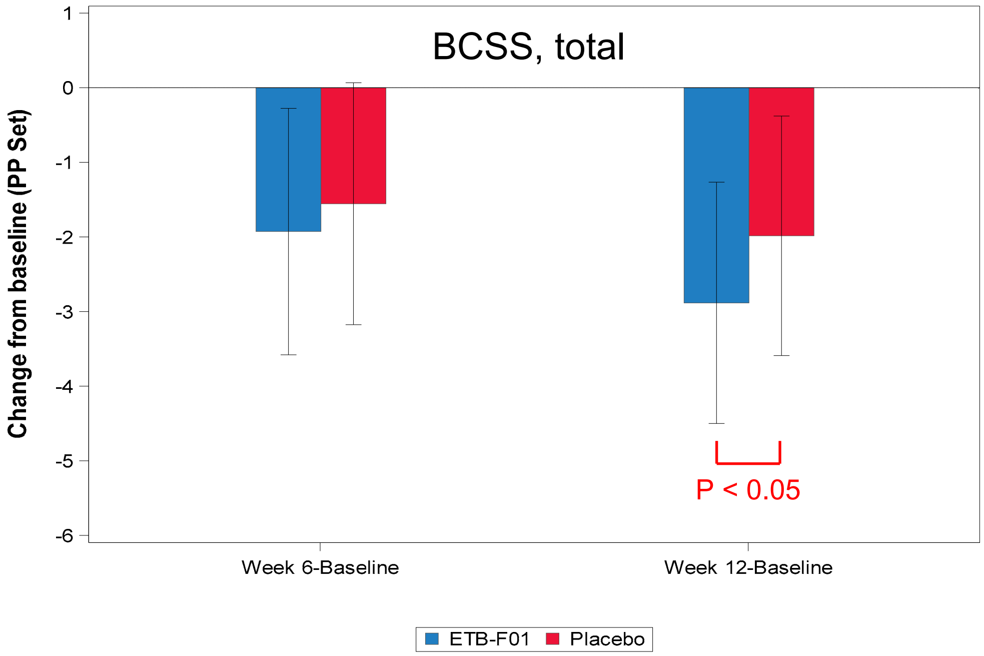

Figure S2. Mean change from baseline in BCSS total score in the full analysis set.

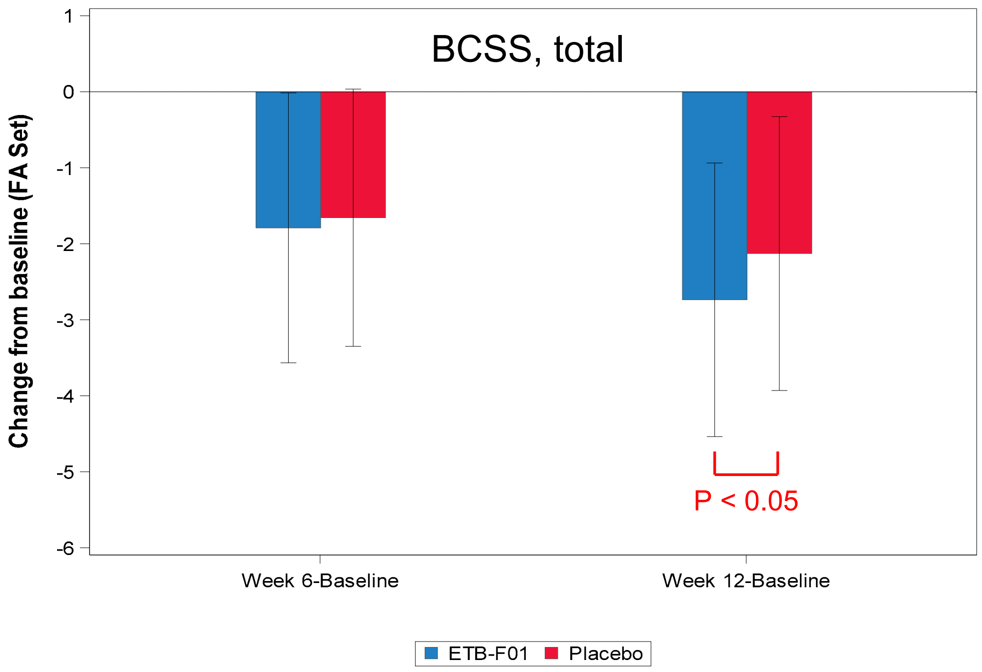

Figure S3. Mean change from baseline in BCSS breathlessness score in the per-protocol set.

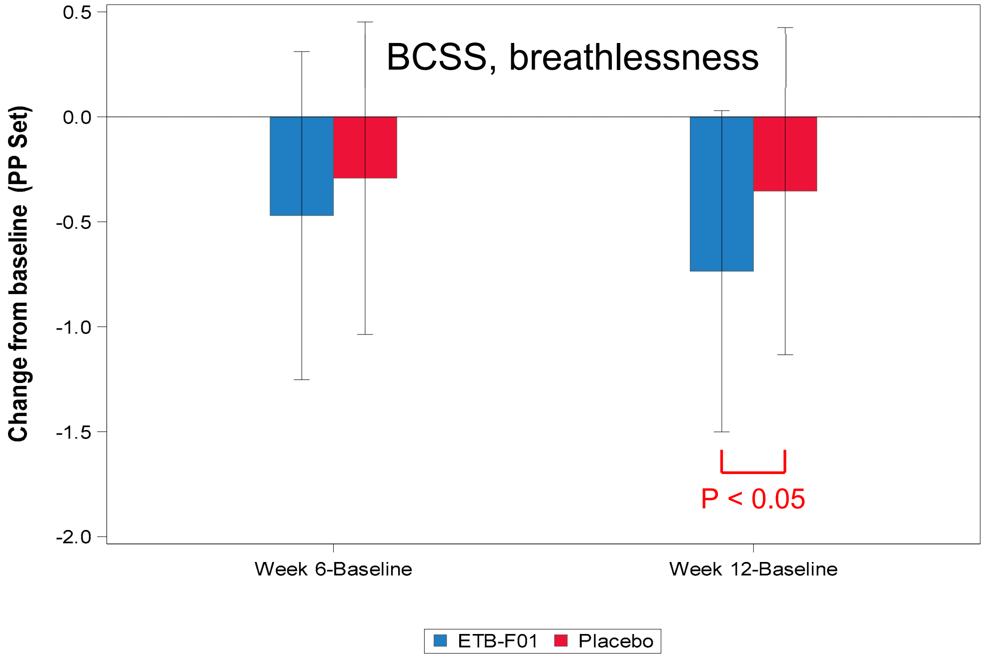

Figure S4. Mean change from baseline in BCSS breathlessness score in the full analysis set.

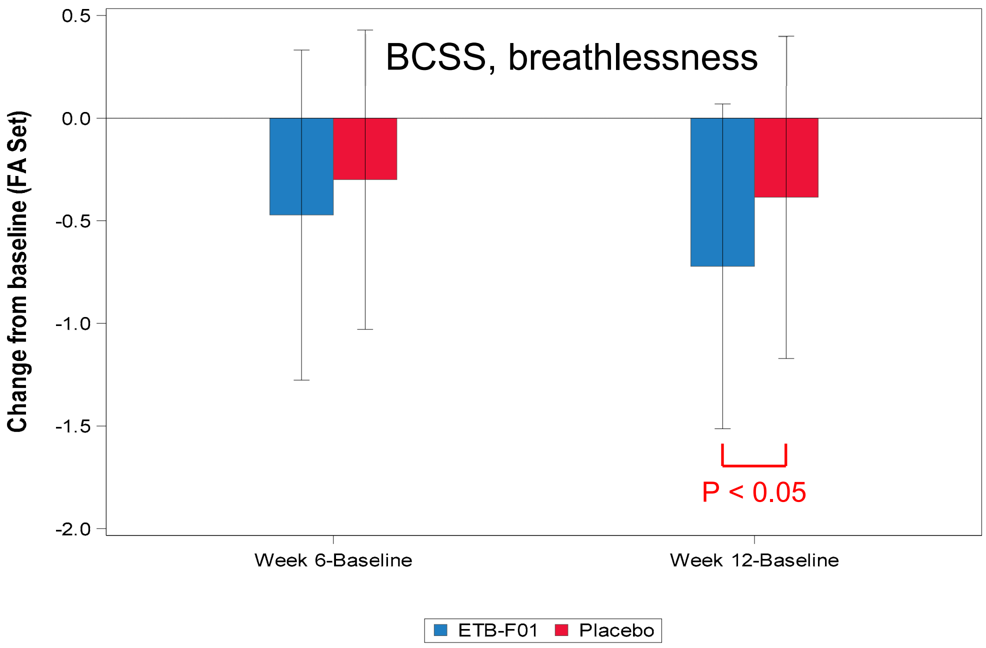

Figure S5. Mean change from baseline in BCSS cough score in the per-protocol set.

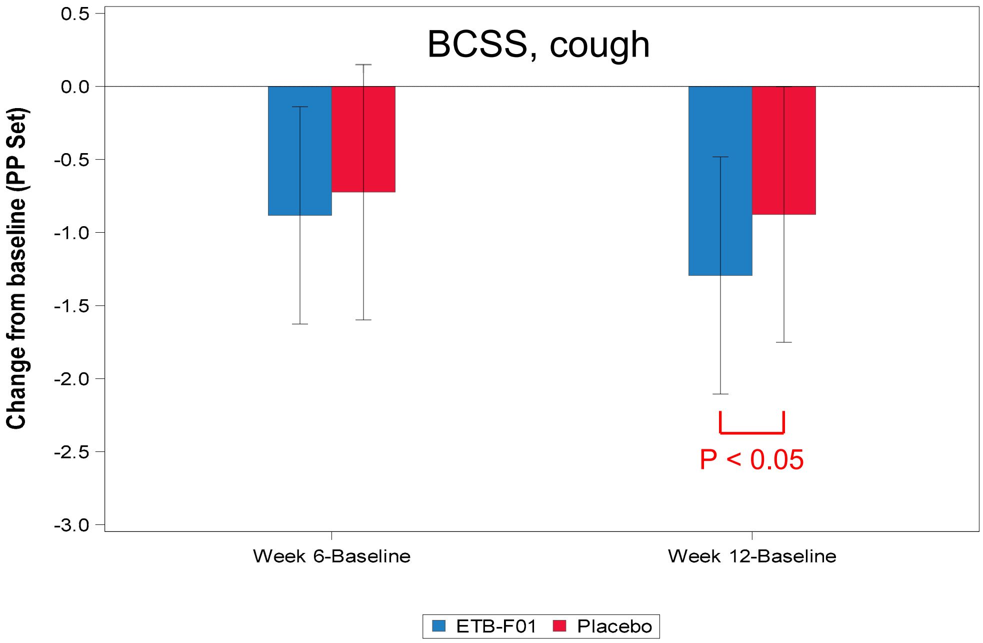

Figure S6. Mean change from baseline in BCSS cough score in the full analysis set.

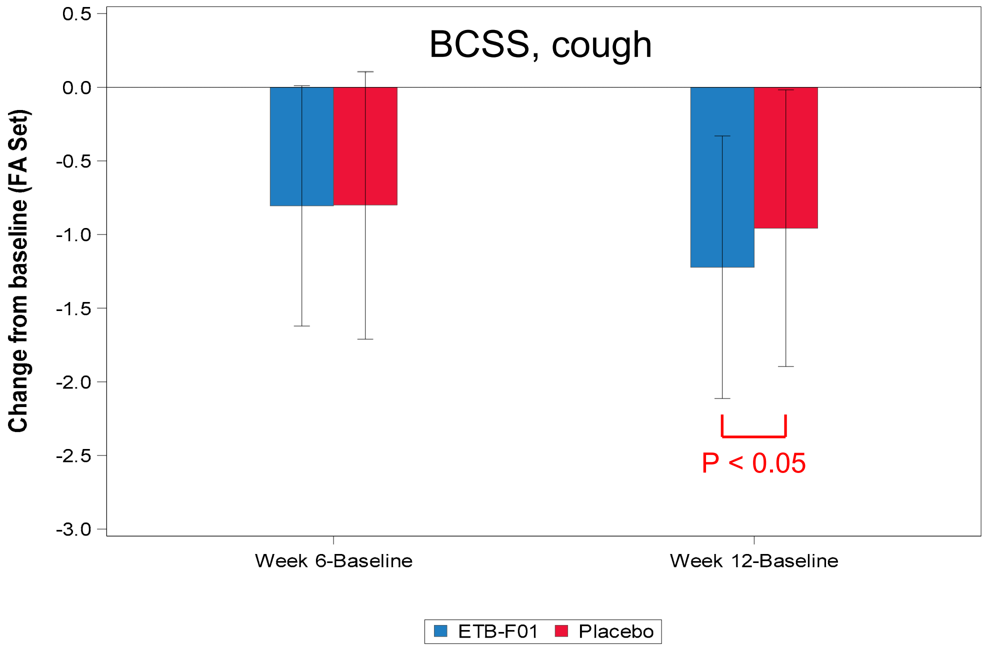

Figure S7. Mean change from baseline in BCSS sputum score in the per-protocol set.

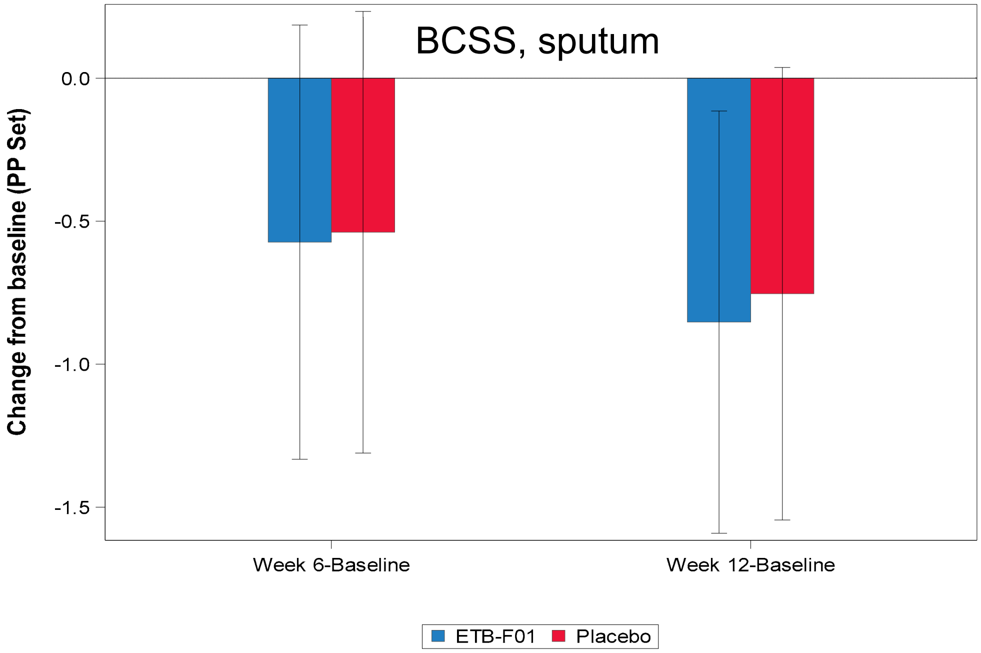

Figure S8. Mean change from baseline in BCSS sputum score in the full analysis set.

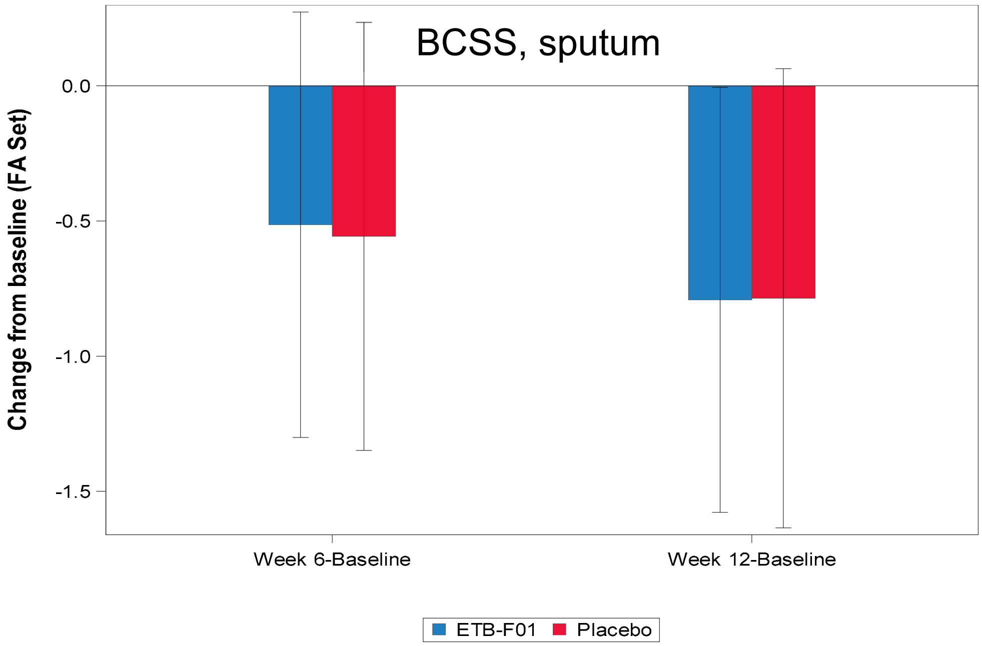

Table S2. mMRC and SGRQ.

| Outcome | ETB-F01 (n=68) | | Placebo (n=65) |
|---|---|---|---|
| **mMRC** | | | |
| At week 12, mean ± SD | 0.6±0.6 | | 0.6±0.5 |
| Change from baseline to week 12, mean ± SD | -0.6±0.8 | | -0.6±0.6 |
| P-value within groups | <0.001 | | <0.001 |
| P-value between groups | | 0.961 | |
| Change from baseline to week 12, LS mean ± SE | -0.5±0.2 | | -0.5±0.2 |
| Difference in change from baseline to week 12, LS mean, (95% CI) | | 0.01 (-0.2, 0.3) | |
| P-value[a] | | 0.937 | |
| **SGRQ** | | | |
| At week 12, mean ± SD | 7.2±11.3 | | 12.3±13.9 |
| Change from baseline to week 12, mean ± SD | -15.4±17.5 | | -13.7±15.9 |
| P-value within groups | <0.001 | | <0.001 |
| P-value between groups | | 0.944 | |
| Change from baseline to week 12, LS mean ± SE | -10.6±4.4 | | -9.6±3.9 |
| Difference in change from baseline to week 12, LS mean, (95% CI) | | -1.0 (-6.8, 4.9) | |
| P-value[a] | | 0.745 | |

CI, confidence interval; LS, least squares; mMRC, Modified Medical Research Council Dyspnea Scale; SD, standard deviation; SE, standard error; SGRQ, St. George's Respiratory Questionnaire. [a] p-value for ANCOVA-adjusted smoking history.

Table S3. VAS.

| Outcome | ETB-F01 (n=68) | Placebo (n=65) |
|---|---|---|
| **VAS breathlessness, mm** | | |
| At week 12, mean ± SD | 6.2±11.0 | 9.3±12.4 |
| Change from baseline to week 12, mean ± SD | -18.5±20.3 | -15.4±18.5 |
| P-value within groups | <0.001 | <0.001 |
| P-value between groups | 0.392 | |
| Change from baseline to week 12, LS mean ± SE | -23.5±5.1 | -19.6±4.5 |
| Difference in change from baseline to week 12, LS mean, (95% CI) | -3.9 (-10.7, 2.9) | |
| P-value[a] | 0.258 | |
| **VAS cough, mm** | | |
| At week 12, mean ± SD | 8.0±10.3 | 15.2±15.5 |
| Change from baseline to week 12, mean ± SD | -24.1±20.0 | -18.0±18.9 |
| P-value within groups | <0.001 | <0.001 |
| P-value between groups | 0.074 | |
| Change from baseline to week 12, LS mean ± SE | -28.6±5.1 | -21.9±4.5 |
| Difference in change from baseline to week 12, LS mean, (95% CI) | -6.8 (-13.6, 0.0) | |
| P-value[a] | 0.051 | |
| **VAS sputum, mm** | | |
| At week 12, mean ± SD | 11.1±14.3 | 14.9±16.1 |
| Change from baseline to week 12, mean ± SD | -23.5±20.9 | -23.3±21.5 |
| P-value within groups | <0.001 | <0.001 |
| P-value between groups | 0.973 | |
| Change from baseline to week 12, LS mean ± SE | -28.1±5.6 | -27.1±4.9 |
| Difference in change from baseline to week 12, LS mean, (95% CI) | -1.0 (-8.4, 6.5) | |
| P-value[a] | 0.798 | |

CI, confidence interval; LS, least squares; SD, standard deviation; SE, standard error; VAS, Visual Analog Scale. [a] p-value for ANCOVA-adjusted smoking history.

Table S4. Blood test results.

| Variables | ETB-F01 (*n* = 77) | | | P-value[1] | Placebo (*n* = 73) | | | P-value[1] | ETB-F01 vs. Placebo P-value[2] |
|---|---|---|---|---|---|---|---|---|---|
| | Baseline | 12 Weeks | Mean Change | | Baseline | 12 Weeks | Mean Change | | |
| Hematological parameters | | | | | | | | | |
| WBC ($10^3$/μL) | 6.21 ± 1.58 | 6.08 ± 1.43 | -0.08 ± 1.20 | 0.58 (P) | 6.13 ± 1.72 | 6.08 ± 1.43 | -0.01 ± 1.56 | 0.60 (S) | 0.99 (W) |
| RBC ($10^6$/μL) | 4.52 ± 0.42 | 4.49 ± 0.40 | 0.00 ± 0.20 | 0.85 (S) | 4.56 ± 0.41 | 4.54 ± 0.46 | -0.03 ± 0.25 | 0.38 (P) | 0.57 (W) |
| Hb (g/dL) | 13.50 ± 1.30 | 13.43 ± 1.24 | 0.01 ± 0.62 | 0.92 (P) | 13.50 ± 1.40 | 13.43 ± 1.47 | -0.07 ± 0.66 | 0.36 (P) | 0.46 (T) |
| Hct (%) | 40.54 ± 3.55 | 40.27 ± 3.29 | -0.06 ± 2.03 | 0.74 (S) | 40.67 ± 3.39 | 40.34 ± 3.87 | -0.38 ± 1.98 | 0.12 (P) | 0.38 (W) |
| Platelet ($10^3$/μL) | 259.06 ± 47.07 | 254.66 ± 56.33 | -3.77 ± 30.00 | 0.31 (S) | 270.12 ± 52.27 | 263.74 ± 54.89 | -6.71 ± 28.13 | 0.05 (P) | 0.35 (W) |
| Neutrophil (%) | 55.28 ± 8.62 | 55.03 ± 8.85 | 0.16 ± 8.18 | 0.59 (S) | 56.61 ± 8.99 | 56.57 ± 9.63 | -0.26 ± 8.88 | 0.81 (P) | 0.95 (W) |
| Lymphocyte (%) | 35.08 ± 7.89 | 35.22 ± 8.49 | -0.15 ± 6.83 | 0.73 (S) | 33.90 ± 7.88 | 33.34 ± 8.68 | -0.37 ± 7.92 | 0.70 (P) | 0.66 (W) |
| Monocyte (%) | 6.59 ± 1.97 | 6.77 ± 1.95 | 0.14 ± 1.73 | 0.51 (P) | 6.20 ± 1.68 | 6.50 ± 1.82 | 0.34 ± 1.35 | 0.01 (S) | 0.35 (W) |
| Eosinophil (%) | 2.12 ± 1.70 | 2.04 ± 1.07 | -0.17 ± 1.62 | 0.53 (S) | 2.39 ± 2.18 | 2.73 ± 2.09 | 0.35 ± 1.69 | 0.06 (S) | 0.47 (W) |
| Basophil (%) | 0.56 ± 0.32 | 0.60 ± 0.29 | 0.03 ± 0.26 | 0.23 (S) | 0.60 ± 0.32 | 0.62 ± 0.32 | 0.02 ± 0.24 | 0.58 (P) | 0.60 (W) |
| **Biochemical parameters** | | | | | | | | | |
| AST (GOT)(IU/L) | 20.66 ± 5.49 | 21.86 ± 7.42 | 1.52 ± 6.82 | 0.10 (S) | 20.23 ± 5.05 | 20.00 ± 6.48 | 0.04 ± 6.40 | 0.34 (S) | 0.07 (W) |
| ALT (GPT)(IU/L) | 17.81 ± 10.48 | 19.20 ± 14.99 | 2.54 ± 13.12 | 0.15 (S) | 17.25 ± 9.23 | 16.65 ± 8.23 | -0.59 ± 6.09 | 0.51 (S) | 0.14 (W) |
| Total Cholesterol (mg/dL) | 195.60 ± 28.79 | 194.01 ± 31.02 | -0.07 ± 20.85 | 0.83 (S) | 196.13 ± 36.72 | 200.16 ± 39.46 | 2.24 ± 19.56 | 0.36 (P) | 0.79 (W) |
| Glucose (mg/dL) | 95.39 ± 13.81 | 94.96 ± 12.83 | -0.59 ± 16.29 | 0.95 (S) | 96.82 ± 11.75 | 93.68 ± 11.06 | -2.85 ± 14.11 | 0.16 (S) | 0.34 (W) |
| Total Protein (g/dL) | 7.44 ± 0.35 | 7.36 ± 0.36 | -0.07 ± 0.32 | 0.07 (P) | 7.47 ± 0.39 | 7.41 ± 0.44 | -0.06 ± 0.39 | 0.07 (S) | 0.63 (W) |
| BUN (mg/dL) | 12.26 ± 3.08 | 12.58 ± 3.50 | 0.13 ± 2.92 | 0.71 (P) | 12.84 ± 3.68 | 13.24 ± 2.99 | 0.38 ± 3.55 | 0.38 (P) | 0.65 (T) |
| Creatinine (mg/dL) | 0.71 ± 0.14 | 0.73 ± 0.14 | 0.02 ± 0.11 | 0.20 (S) | 0.71 ± 0.14 | 0.74 ± 0 .14 | 0.02 ± 0.09 | 0.14 (S) | 0.91 (W) |
| Uric acid (mg/dL) | 4.51 ± 1.03 | 4.59 ± 1.04 | 0.12 ± 0.65 | 0.14 (P) | 4.69 ± 1.19 | 4.95 ± 1.45 | 0.23 ± 0.76 | 0.02 (P) | 0.35 (T) |
| Ca (mg/dL) | 9.53 ± 0.41 | 9.54 ± 0.35 | 0.01 ± 0.33 | 0.99 (S) | 9.58 ± 0.38 | 9.51 ± 0.39 | -0.06 ± 0.36 | 0.20 (P) | 0.32 (W) |

The values are presented as the mean ± SD.

[1] Compared within groups; P-value for paired t-test (P) or Wilcoxon signed rank test (S).

[2] Compared between groups; P-value for two-sample t-test (T) or Wilcoxon rank sum test (W).

Table S5. BCSS score according to sex groups.

| Variables | 12 Weeks | Mean Change | P-value[1] | 12 Weeks | Mean Change | P-value[1] | ETB-F01 vs. Placebo P-value[2] |
|---|---|---|---|---|---|---|---|
| **Females** | **ETB-F01 (*n* = 55)** | | | **Placebo (*n* = 46)** | | | |
| BCSS total score | 1.38±1.24 | -2.93±1.61 | < 0.0001 | 2.37±1.61 | -1.89±1.55 | < 0.0001 | 0.0017 |
| BCSS breathlessness score | 0.35±0.55 | -0.75±0.75 | < 0.0001 | 0.70±0.63 | -0.28±0.81 | 0.0219 | 0.0067 |
| BCSS cough score | 0.49±0.60 | -1.31±0.84 | < 0.0001 | 0.91±0.72 | -0.87±0.83 | < 0.0001 | 0.0156 |
| BCSS sputum score | 0.55±0.57 | -0.87±0.72 | < 0.0001 | 0.76±0.67 | -0.74±0.80 | < 0.0001 | 0.4066 |
| **Males** | **ETB-F01 (*n* = 13)** | | | **Placebo (*n* = 19)** | | | |
| BCSS total score | 2.31±1.65 | -2.69±1.70 | < 0.0001 | 2.11±2.47 | -2.21±2.10 | < 0.0001 | 0.5827 |
| BCSS breathlessness score | 0.62±0.65 | -0.69±0.85 | 0.0128 | 0.47±0.51 | -0.53±0.70 | 0.0040 | 0.5419 |
| BCSS cough score | 0.85±0.69 | -1.23±0.73 | < 0.0001 | 0.84±0.69 | -0.89±0.99 | 0.0010 | 0.4108 |
| BCSS sputum score | 0.85±0.69 | -0.77±0.83 | 0.0060 | 0.79±0.63 | -0.79±0.79 | 0.0004 | 0.8432 |

The values are presented as the mean ± SD.

[1] Compared within groups; P-value for paired t-test (P).

[2] Compared between groups; P-value for two-sample t-test (T).
